# Preventive and Therapeutic Vaccines against Human Papillomaviruses Associated Cervical Cancers

**Published:** 2012

**Authors:** Khadem Ghaebi Nayereh, Ghaeb Khadem

**Affiliations:** 1 *Women’s Health Research Centre, Mashhad University of Medical Sciences, Mashhad, Iran*; 2 * Microbiology and Virology Research Centre, Mashhad University of Medical Sciences, Mashhad, Iran*

**Keywords:** Cervical cancer, Human papillomavirus, Preventive vaccine, Therapeutic vaccine

## Abstract

Cervical cancer is, globally known to be, one of the most common cancers among women especially in developing countries. More than 90% of cervical cancers are associated with high-risk human papillomaviruses (HPVs) particularly HPV types 16 and 18. Two major strategies have been developed for prevention and treatment of cervical cancer and other HPV-associated malignancies; the first one is based on HPV virus-like particles (VLPs) containing HPV structural proteins. VLP based vaccines can induce genotype specific virus neutralizing antibodies for preventing HPV infections. The other strategy is based on HPV early genes especially E6 and E7 for eliminating the established HPV infections; therefore they are classified as HPV therapeutic vaccines. This article reviews the preventive and therapeutic vaccines against HPV infections and cervical cancer.

## Introduction

Cervical cancer ranks second in the list of the most common cancers and the third in the list of cancer death in women worldwide (1, 2). Globally, 493,000 cases of cervical cancer are recognized each year and cervical cancer brings about the death of 274,000 women annually (3). World Health Organization (WHO) introduced cervical cancer as the first cancer which is entirely caused by infection (4). This specification of cervical cancers makes them appropriate for designing the therapeutic vaccines or molecular cancer treatment technique by targeting the infected cells which express the specific viral antigens. Since persistent infection of human papillomaviruses (HPVs) is the main risk factor for development of cervical cancer and the virus DNA has been distinguished in 99.7% of cervical cancer specimens, development of the new vaccines may provide an opportunity for protecting and/or treating the cancer by attacking the involved viral antigens (1, 5). The rate of cervical cancer has been reduced in countries that implement screening programs but most developing countries lack the screening. Cervical cancer rate is 450,000 cases per year in developing countries that is more than 80% of cervical cancer occurrence (4, 6). Although systemic cervical screening programs based on cervical cytology have decreased mortality of cervical cancer in developed countries, there are multiple limitations of cervical screening programs such as poor sensitivity of cervical cytology, anxiety and morbidity of screening programs for large numbers of screened women, poor acceptance by some communities and no or bad predictive value for adenocarcinoma (7). In addition, cervical screening program is not easily available in third world countries where the possibility of cervical cancer incidence and mortality are high (8). In addition to referrals and treatment of precancerous lesions, screening strategies is expensive to perform. Also, the current treatments show variable cure rates and recurrence has frequently occurred (6, 9); thus, the significance of prophylactic and therapeutic vaccines is revealed in its relation to reducing the morbidity and mortality due to cervical cancers. This review summarizes the recent advances on preventive and therapeutic HPV vaccines.

## Cervical cancer and HPV

Cervical cancer is the principle cause of women cancer deaths in developing countries (10, 11). In different studies, the incidence of cervical cancer varies from 4.2 per 100,000 to 54.6 per 100,000 (12-14). HPV infection is currently the most common sexually transmitted disease worldwide (15). Regarding the high prevalence of HPV infection and its strong ties association with cervical cancer, there has been a need to develop preventive and therapeutic vaccines. Nowadays, more than 200 HPV genotypes have been molecularly identified and about 50 types can infect the genital mucosa via sexual transmission (1, 16-18). Genital types of HPV are classified into low- or high-risk types depending on the clinical prognosis of the lesions they produce and their association with cervical cancer and precancerous lesions (1, 16, 19), and approximately 16 types are highly carcinogenic for which different vaccines should be designed (18). Low-risk HPV types include types 6, 11, 42, 43, 44 and high-risk HPV types are 16, 18, 31, 33, 34, 35, 39, 45, 51, 52, 56, 58, 59, 66 ,68 and 70. There are a few HPV types in high-risk group that are less frequently found in cancers but are often found in squamous intraepithelial lesions which some authors refer to as intermediate-risk (11, 20). A strong interrelationship exists between high-risk HPVs and the development of ano-genital intraepithelial neoplasia conditions (21). HPV types 16 and 18 are the most common members of the high-risk HPVs and account for 70% or more of all cervical cancers worldwide (1, 16, 21). HPV-16 can be detected in about half of all invasive cervical cancers and is regarded as the most prevalent high-risk HPV in cervical cancer types among Iranian women (21, 22). According to the high prevalence of HPV type 16 in our country, new designed vaccines should be at least consisted of this virus type to cover more infected people. HPV-18 is the second most prevalent type and is found in about 16% of cervical cancer cases (22). E6 and E7 are two major oncoproteins of high-risk HPVs (15, 21). E6 is a multifunctional protein and affects growth and proliferation of infected cells. Other functions of the E6 include cell immortalization, transformation, tumor formation and apoptosis (12, 23). The E6 protein binds to p53 which is a tumor suppressor protein and eliminates the p53 protein by facilitating its degradation (21, 24, 25). The high-risk HPVs’ E7 protein is a viral oncoprotein that binds to retinoblastoma protein (pRb) and also decreases the transcriptional activity of p53 (21, 26). The continuous expression of E6 and E7 genes is required for inactivation of normal function of p53 and pRb tumor suppressor genes, genomic instability, suppression of apoptosis and inhibition of cyclin-dependent kinase inhibitors and cellular antibodies; E6 and E7 functions lead to accumulation of genetic changes resulting in malignancies and invasive cancers (12, 27-30). The type of cervical lesions indicates the degree of E6 and E7 expression; E6 and E7 are expressed at low levels in basal cells and at higher levels in the upper layers of epithelium in low-grade lesions, but in high-grade lesions, they are expressed at high-levels throughout the epithelium (29). Regarding the above explanation, the E6 and E7 genes of HPVs are ideal targets for designing and constructing new therapeutic vaccines. 

## HPV structure and genome organization

Papillomaviruses (PVs) are small, non-enveloped, epitheliotropic DNA viruses that are 52-55 nanometers in diameter and consist of a circular double-stranded DNA approximately 8000 base pairs (bp) in size (1, 16, 31, 32). The coding strand of the viral DNA includes about 10 open reading frames (ORFs) that are classified as early (E) and late (L) ORFs based on their location within the virus genome (1, 31). The early region encodes for viral replication and cellular transformation (1, 32). The late region includes L1 and L2 ORFs that encodes the viral structural proteins and express only in productive infected cells. These viral capsid proteins are exposed to the host immune system and are the main viral antigens for neutralizing antibodies; therefore, they can be used as appropriate targets for development of preventive HPV vaccines. ORFs L1 and E6 are separated by a region of approximately 1 kilobase pair (kbp) that contain no ORF and are referred to as a non-coding region (NCR), long control region (LCR) or upstream regulatory region (URR) (31). This region contains promoter and enhancer DNA sequences essential to regulate viral replication and transcription (32). 

## HPV infection and oncogenesis

The primary way of HPV transmission is by skin to skin contact and more than 50% of all sexually active adults are exposed to it at least once in their lifetime (33). The clinical spectrum of HPV infections ranges from asymptomatic infections to benign warts (primarily low-risk HPV types 6 and 11) to invasive malignancy (6, 11, 15, 33-37). Most HPV infections can be treated but some infections with high-risk HPVs can progress to cancers of cervix, vulva, vagina, anus, penis, breast, oropharynx and oral cavity (38-41). More than 70% of cervical cancers are related to risky HPV types 16 and 18 (6, 15). The primary risk factor for HPV infections is sexual activity (29). The relation between HPV and cervical cancer is 10 times more than the relation between smoking and lung cancer (42). As it was mentioned before there is a high association between HPV and cervical cancer; thus, administration of therapeutic and preventive vaccines against HPV will be beneficent to highly decrease the contingency of cervical cancer. Other risk factors are smoking, oral contraceptive use, parity, herpes simplex virus (HSV), and *Chlamydia trachomatis* infections (6, 29, 43). In addition, increasing in the number of lifetime sexual partners and early onset of sexual activity is the other risk factors for HPV infections (28, 33). For progression of HPV infection and development of cancer, several steps occurs including overcoming host immune responses, possible integration of HPV DNA into the host chromosome and accumulation of the resulting mutations within the infected cells (29). 

## Immune responses to HPV

HPV infection and virus life cycle occur in epithelial cells. Infectious viruses are released through desquamating cells; therefore, exposure of immune system to viral antigens is limited (31, 44). Both innate and adaptive immunity are engaged in HPV infection clearance. Humoral and cellular immune responses against HPV infection are elicited (34). T-cell immune responses are very important for regression once the host has been infected and humoral immunity is most likely involved to prevent the spread of infection within the host and re-infection (31); therefore, in designing a new vaccine, it is important to note that a therapeutic vaccine should be able to induce cell mediated immunity while a preventive vaccine should be able to excite humoral immune responses. Serum antibodies against different HPV proteins are produced (34). Antibodies against L1 epitopes are neutralizing type-specific antibodies. An important feature of L1 proteins is their self-assemble properties and producing virus-like particles (VLPs) which are important steps in development of HPV preventive and chimeric vaccines (44). Natural HPV infection is cleared by specific cell - mediated immune responses (45). Both CD4^+^ and CD8^+^ T-cell responses to HPV E6 and E7 oncoproteins possess a role in modulating of HPV infections and diseases. HPV can induce the mucosal immune responses; however, their role in resolving the infection and protection from infection is unknown (6). There are several mechanisms for evading the HPV from immune responses. HPV only infects the basal layer cells and viral replication and assembly occur only in fully differentiated cells; therefore, HPV avoids the immune system of the host. Moreover, the humoral and cellular immune responses against HPV infection are very poor (28). In HPV infection, pro-inflammatory signals which activate the dendritic cells are not elicited due to non-lytic properties of HPV propagation steps. Furthermore, the viral proteins are accumulated in nucleus of infected cells and are not secreted. The L1 and L2 capsid proteins are expressed in terminally differentiated outer layers that have little contact with immune responses at epithelium (6). HPV escapes immune recognition via several mechanisms such as down-regulation of expression of TLR, MCP1, IL8, blocking the function of IFN-α and repression of MHC class II [more information about all mechanisms used by HPV to escape the host immune responses are reviewed by Kanodia *et al *(46)]. 

## Vaccines for HPV

High-risk HPV types 16, 18, 31 and 45 are common causes of cervical adenocarcinoma that is extended more rapidly than squamous cell carcinoma and cannot be detected easily by cervical screening (2). In addition, it is often not able to detect the adenocarcinoma and is only capable of detecting neoplastic changes after their occurrence; therefore, a prophylactic vaccine is valuable in early stages (41, 47). Prevention of HPV-16 and 18 infections by prophylactic vaccination in association with cervical screening programs may be of great help to further improvement of cervical cancer prevention (7). HPV vaccine development and determination of vaccine efficacy are difficult because of the lake of animal models for HPV infections and the lake of cell culture for HPV propagation in order to develop killed or attenuated viral vaccines (48). Current viral vaccines against HPV that are licensed for use in humans are prophylactic vaccines which prevent the viral infection via induction of virus-neutralizing antibodies. Prophylactic viral vaccines should be able to induce the neutralizing antibodies as well as to recall the specific memory cells for future viral infections (4). The aim of preventive vaccines is to generate strong type-specific immune responses against the most prevalent high-risk HPV types, especially HPV-16 and 18. It can also be of help for preventing cervical disease outcomes associated with these viruses. Infections with HPV-16 and HPV-18 are a main risk factor for developing of a cervical cancer; therefore, HPV vaccination for preventing cervical cancer is primarily aimed at HPV-16 and HPV-18. The preventive vaccines that are recently administered contain just these two types of high-risk HPVs; therefore, they are not able to protect vaccinated subjects against the other high-risk types of the virus. Furthermore, the elimination of two high-risk types of the virus by vaccination may lead to gradual replacement and increase of the other cancerogenic types resulting in the escalation of cervical cancer occurrence again; therefore, there is a need to develop new preventive vaccines containing all high-risk types of HPVs. Moreover, the present vaccines screening program should be carried out continuously for detecting the development of high-risk types in order to design other preventive vaccines. The evaluation of all recent HPV vaccine efficacies has been primary done by effects of a vaccine on preventing CIN associated with HPV-16/18 DNA in the precancerous tissue biopsy; however, this method is not the only useful way to assess cross-protection on prevention of cervical cancer (22) 

 Another type of HPV vaccines are therapeutic vaccines that should be able to develop the immune responses for eliminating the cells already infected with the virus. These kinds of vaccines are designed to prime the antigen-specific T-cell mediated mechanisms for controlling and eliminating the viral infections (4). HPV early proteins especially E6 and E7 make up the basic targets for development of therapeutic vaccines because they are expressed early in viral infection and are essential for transformation of the infected cells. In addition, these genes are continuously expressed in HPV infected cells but not in normal cells; their expression is needed for keeping the malignant state of the infected cells. Furthermore, they are able to generate T-cell mediated immune responses to eliminate the infected cells (1). 

## HPV preventive vaccines

Neutralizing antibodies are the main keys for prevention of viral infections (4). Natural HPV infections induce low antibody concentrations and women with an acquired HPV infection remain at risk for a new infection with the same type of HPV (49). The expression of HPV L1 protein in eukaryotic cells results in the VLPs that mimic the natural structure of the virus (4, 28, 44). Early studies to develop the HPV vaccines were done on animal models (28, 34, 48). Previous studies showed the immunization of animals with recombinant L1 HPV VLPs primed immune system to generate neutralizing antibodies against the wild-type virus and protected vaccinated animals against experimental infection with the homologous animal papillomavirus (4, 44, 48, 50, 51). In addition, passive transfer of sera from HPV VLP-vaccinated mice to non-vaccinated mice showed protection which mediated likely via neutralizing antibodies; furthermore, previous studies showed that protection required intact VLPs containing conformational epitopes while denatured VLPs showed no protective immune effects (48). Several studies showed that HPV VLPs are highly immunogenic and antibody titers after immunization with HPV VLPs are 80- to 100-fold higher than those measured following natural infection. Furthermore, the mean of antibody titers remain 10- to 16-fold higher in vaccinated group than in those made by natural HPV infection after 18 months (51). 

 In the first clinical trial for HPV vaccine, a monovalent HPV-16 L1 VLPs vaccine was used in humans and the results showed 100% vaccine efficacy against all grades of CIN (28, 34, 52). This vaccine was highly immunogenic and well tolerated (34).

Nowadays, there are two preventive HPV vaccines: the quadrivalent and bivalent vaccines (17). Gardasil^®^ is a quadrivalent vaccine containing non-infectious recombinant L1 VLPs of low-risk HPV types 6 and 11, and high-risk HPV types 16 and 18 mixed with an aluminum-containing adjuvant (6, 17). The L1 protein is expressed in yeast (*Saccharomyces cerevisiae*) and the proteins self-assemble into non-infectious VLPs (34). This vaccine was approved by United States Food and Drug Administration (FDA) in June 2006 for women aged 9-26 years (34, 53-55). The vaccine is administered intramuscularly as three separate doses (0.5 ml). Each 0.5 ml dose contains 20 µg HPV-6 L1 protein, 40 µg HPV-11 L1 protein, 40 µg HPV-16 L1 protein and 20 µg HPV-18 L1 protein (34). The Gardasil^®^ is expected to be highly effective in preventing HPV-16/18 infections, CIN2 or CIN3, cervical cancer and also in preventing HPV-6/11 genital warts (33, 56, 57); however, the occurrence of a cervical cancer may take several years and vaccinated individuals should be followed for a long time. Quadrivalent vaccine and placebo were used for vaccination of 12167 women aged 16-26 years (17). Efficacy of Gardasil^®^ has been observed in randomized, multi-center, double-blind, placebo-controlled phase II and III studies (33). The vaccine efficacy was 44% for HPV-16/18 related CIN2/3 and adenocarcinoma in situ (17). Moreover, it was 98% effective for prevention of HPV-16/18 related high-grade cervical precancerous lesions (17, 58, 59), but it showed only 48% efficacy in all women randomized at entry (58). The influencing factors that lead to low efficacy of the vaccine should be determined and the possible ways to increase the vaccine efficacy and safety also should be considered. In addition, the efficacy of the vaccine for vaccinated subjects of different countries should be evaluated. Gardasil^®^ vaccine appears to be well tolerated. In the Gardasil^®^ trial, injection side effects such as erythema, pain, and swelling were the most common side effects. It was more frequently observed in vaccinated group (87%) than in the placebo group (77%). Also, systemic adverse events were reported by a similar proportion of both groups. No deaths secondary to vaccine were reported in the trial (34). Pregnant women were excluded from HPV vaccine trials, but some women became pregnant during the trial. In the phase III studies of the quadrivalent vaccine, 1396 women in the vaccine group and 1436 women in the placebo group became pregnant. No statistical differences were observed in the ratio of spontaneous abortions, late fatal death and overall congenital anomalies between vaccine-injected and placebo-injected groups; however, subgroup analysis showed five cases of congenital anomalies in vaccine-injected cases that became pregnant within 30 days of a vaccine dose compared with no cases in placebo- injected one (33). Congenital anomalies should be evaluated for longer period in larger population for detecting the rate of risks after administration of preventive vaccines. Several studies suggest that quadrivalent HPV L1 VLP vaccine, among adolescents will be safe and effective and high titers of antibody are produced after vaccination at ages of 11-12 years; however, it can be administered to female as young as 9 years old and female aged above 12. Vaccination with this vaccine is also recommended for females before potential exposure to HPV through sexual contact; however, females with probable exposure to HPV should also be vaccinated (34). Totally, Gardasil^®^ shows an excellent safety profile and type-specific protection and is a highly immunogenic HPV prophylactic vaccine (60); however, it should be noted that this vaccine contains only four HPV types and it is not effective for prevention purposes for the other HPV types.

 Cervarix^™^ is a bivalent vaccine containing L1 VLPs of two most prevalent HPV genotypes 16 and 18 expressed in an insect cell system (6, 61). The vaccine contains aluminum hydroxide with deacylated monophosphoryl lipid A (ASO4) adjuvant (6, 62). Cervarix^™^ is administered as a three-dose vaccine regime at 0, 1 and 6 months (62). Each dose contains 20 µg HPV-16 L1 protein and 20 µg HPV-18 L1 protein (63, 64). The vaccine was approved in October 2009 by the Advisory Committee on Immunization Practices (ACIP) for usage in Australia and Europe (54, 65). Cervarix^™^ has been shown to induce long-lasting antibody responses and to protect against CIN, cervical infection and persistent infection which are related to the vaccine HPV types 16 and 18 (22); therefore bivalent vaccine Cervarix^™ ^is useful for protection against CIN2/3 and cervical cancer but not genital warts (33). In addition, Cervarix^™ ^has been shown an evidence of protection against oncogenic HPV types 45 and 31 that are phylogenetically related to HPV-16 and HPV-18 (60). Bivalent vaccine was used for vaccination of 18644 women aged 15-25 years. Its efficacy was 90.4% against HPV-16/18 related CIN2/3 lesions in HPV-16/18 seronegative and DNA negative at day 0 of the trial (17). Efficacy of Cervarix^™^ vaccine has been also established in randomized, multi-center, double-blind, placebo-controlled phase II and III studies (33). Cervarix^™^ vaccine has shown long-term efficacy, high and sustained immunogenicity and desirable safety up to 6.4 years (49). Furthermore, the vaccine has shown evidence of cross-protection against infection with two non-vaccine HPV types 45 and 31 that are phylogenetically related to HPV-16 and HPV-18 (50, 65). HPV types 16, 18, 45 and 31 are responsible for about 80% of cervical cancers worldwide (65, 66). As described before, the Cervarix^™ ^vaccine like Gardasil^®^ vaccine contains limited types of HPVs and cannot protect vaccinated subjects against the other HPV types. Cervarix^™^ vaccine also appears to be safe and well tolerated in the phase IIb studies of the vaccine. Similar to Gardasil^®^ vaccine, its injection site adverse events such as pain, redness and swelling were reported as more common in vaccinated group than placebo group. Systemic adverse experiences were similar in both groups. There were no deaths in the trial considered to be secondary to the vaccine (34). At the time of interim analysis of the bivalent vaccine phase III study, 665 women in vaccine group and 685 women in placebo group became pregnant and no statistical differences were reported in the rate of abnormal infants (33). Considering the existence of congenital anomalies after Gardasil^®^ vaccination, these anomalies should be evaluated after the Cervarix^™^vaccination as well. 

 Different methods were used for evaluation of virus-specific IgG antibody titers after immunization with two VLP vaccines, Cervarix^™^ and Gardasil^®^. A competitive luminex immunoassay (CLIA) or a competitive radioimmuno assay (CRIA) were used to detect defined type- and epitope-specific antibodies against HPV types 6, 11, 16 and 18 in quadrivalent vaccinated subjects and enzyme-linked immunosobent assay (ELISA) technique utilizing VLPs as substrate was used to evaluate total serum anti-VLP IgG antibodies in bivalent vaccinated subjects (51). For vaccine formulation, each VLP type is prepared and purified separately and during final steps, the different types are mixed. Different adjuvants are used in Gardasil^® ^and Cervarix^™^ HPV VLP vaccines. An aluminum adjuvant is used for Gardasil^® ^that typically induce a T-helper 2 (Th2) type of immune responses while ASO4 adjuvant containing monophosphospharyl lipid A and aluminum hydroxide is used for Cervarix^™^ that can induce innate immune responses via toll-like receptor molecules and induce a mix of Th1/Th2 differentiation (63). To evaluate the efficacy of Gardasil^® ^and Cervarix^™^ vaccines, further studies need to be performed by administration of the vaccines using the same adjuvant. In addition, other studies should be done using Gardasil^®^ vaccine with ASO4 adjuvant for evaluating the cross-protection effects of the vaccine against other related/unrelated HPV types. 

 A systemic review of randomized controlled trials of prophylactic vaccination against HPV infections showed prophylactic HPV vaccination to be highly effective in preventing HPV infection and precancerous cervical lesions among not previously HPV infected women aged 15-25 years (67). The efficacy of the vaccines, affinity and duration of the produced antibodies, and adverse side effects should be evaluated in vaccinated individuals in other age groups (>25 and <15 yrs). Yet, immunization with HPV VLPs induces type-specific antibodies against vaccine HPV types. Although some cross-reactivity is observed between HPV-16, 31, 33 and 58 and between HPV-18 and 45, the affinity and duration of these cross-reactive antibodies are currently unknown (51).

 Although prophylactic HPV VLP vaccines open a new approach for preventing HPV infection and related diseases; several studies showed the preventive function of Gardasil^® ^vaccine, none of them have proved the vaccine (either Gardasil^® ^or Cervarix^™^) efficient in preventing the cervical cancer (6, 17); therefore, the new standards for prevention of HPV disease i.e. both preventive vaccination and cervical screening are recommended (68). Preventive vaccination cannot replace cervical screening programs because HPV-16 and HPV-18 are found in approximately 70% of cervical cancers; thus, Gardasil^® ^and Cervarix^™^ do not confer full protection against all HPV high-risk types and cervical cancer; thus, women receiving the vaccines should be aware about the limitations of the vaccines (33, 53). HPV preventive vaccines may be safe and effective; however, they are vaccines for a sexually transmitted disease and their acceptance and marketing for general public may be problematic (28). In addition, duration of protection from HPV infection and long-term safety of the current prophylactic vaccines are still unknown (33). Second generation of polyvalent HPV VLP vaccines will be needed for covering other oncogenic HPV types especially HPV types 45, 31, 56, 52, 35, 33 which are the causes of remaining 20-30% of all cervical cancers (58). Finally, unlike protective effects of VLP preventive vaccines, VLP vaccination failed to generate significant therapeutic effects for established HPV infections (48), and different vaccination strategies are required for development of therapeutic HPV vaccine.

## HPV therapeutic vaccines

As mentioned in previous section, preventive vaccines against HPV are represented to be safe and effective. In addition, they are able to produce high titers of neutralizing antibodies against L1 protein of HPVs; however, they may not be able to treat the established HPV infections and HPV-associated cervical cancers (28, 69). In HPV therapeutic vaccines, stimulation of specific cell-mediated immunity (CMI) against various antigens of HPV is required to eliminate the existing lesions of any grade including invasive cancers, to clear existing HPV infections and to prevent the production and progression of HPV-related lesions (48, 63). As described in previous section, preventive HPV vaccines contain HPV late proteins. To develop the HPV therapeutic vaccines, early HPV proteins such as E1, E2, E5, E6 and E7 proteins have been targeted (28, 69). In HPV therapeutic vaccine studies, E1, E2 and E5 proteins have not been extensively studied because E1 and E2 early proteins of HPV are not expressed continuously in carcinoma and E5 early protein shows limited immunogenicity (63). HPV E6 and E7 early proteins are continuously expressed in most HPV cancers, therefore, they represent good targets for HPV specific therapeutic vaccines (15, 21, 28, 48, 63). Most of the studies used E7 gene as a target for HPV therapeutic vaccine development because E7 is more abundant, more conserved and better immunologically characterized than E6 (48). Different HPV therapeutic vaccines have been developed in experimental systems and some of them are ongoing in human trials; the following sections describe each of them.

## Viral / bacterial live vector vaccines

Different viral vectors have been used for development of HPV viral vector vaccines. Vaccinia virus and adenovirus have been the most common vectors for HPV vaccines. Other forms of viral vectors such as modified vectors, replication deficient viruses, HPV pseudovirions and alphavirus replicon particles have been also used for designing the HPV therapeutic vaccines; since they are able to induce immune responses and have minimal toxicity (69). Vaccinia virus-based vectors have shown several advantages such as high efficiency of infection and high levels of recombinant gene expression (48). Previous studies showed the immunotherapy with vaccinia vectors encoding E6 and/or E7 genes of HPV generated strong CTL activity and anti-tumor responses in pre-clinical studies (48, 70-72). A recombinant vaccinia vector encoding HPV-16 E7 linked to the sorting signal of lysosome-associated protein (LAMP-1) was developed to target E7 to endosomal and lysosomal compartments. This vaccine was administered in mice with E7-expressing tumors and it was able to eliminate the established E7-expressing tumors in the mice. In another study, a recombinant vaccinia virus encoding HPV-16 E7 linked to non-hemolytic portion of listerolysin O (LLO) was developed. This vaccine enhanced CD8^+^ T-cell mediated immune responses and caused the regression of established HPV-16 immortalized tumors in vaccinated mice (48). A phase II trial of a recombinant vaccinia virus-based vaccine, Modified Virus Ankara (MVA), encoding E2 gene was performed in women with CIN2/3. The vaccine was administered via direct injection into the uterine cervix. The data showed that 59% of patients had complete regression and high antibody titers to E2 were detected in all vaccinated patients (73). MVA is a non-replicative attenuated vaccinia virus that is used as a viral vector in several vaccine studies. In a study, a recombinant vaccinia virus consisting of modified E6 and E7 of HPV types 16 and 18 was developed and practiced on women with high-grade vulvar intraepithelial neoplasia. The responding women showed higher levels of CD4^+^ and CD8^+^ responses than non-responders. Unfortunately, none of the vaccinated women had a complete clinical response in this study (74). In a phase II trial in 21 CIN2/3 patients, MVA vaccine encoding E6-E7-IL2 genes was administered. The vaccine showed promising results with about 50% (10 cases) of women having complete regression (6). In several other examinations, recombinant vaccinia vectors containing E6 and/or E7 genes have been developed and preclinical studies proved their high level of generation of CTL activity and anti-tumor responses (70-72, 75). TA-HPV vaccine is a recombinant vaccinia virus encoding E6 and E7 genes of HPV types 16 and 18. Phase I/II clinical trials using TA-HPV vaccine showed development of T-cell immune responses in patients with cervical cancer, CIN3 or early invasive cervical cancer after TA-HPV vaccination (69). In several studies, antigen-specific T-cell responses and anti-tumor effects were shown in vaccination with modified adenovirus vector encoding HPV-16 E6 or E7 (69, 76, 77). A viral-based vaccine used human adenovirus type 5 as a viral vector to express the HPV-16 E7 fused to hepatitis B surface antigen (HBsAg). The vaccine induced high levels of E7-specific humoral and cellular immune responses in vaccinated mice (78). A replication defective adeno-associated virus encoding HPV-16 E7 fused to heat shock protein 70 (HSP 70) was administered as a viral vector vaccine and activation of CD4- and CD8-dependent CTL responses and also anti-tumor effects were indicated in vitro (79). Another group developed recombinant vesicular stomatitis virus (VSV) as viral vector-based therapeutic vaccines to express cotton-tailed rabbit papillomavirus (CRPV) E1, E2, E6 and E7 proteins. Their results culminated in the fact that all four vaccines were effective for eliminating the CRPV disease in vaccinated animals; however, the VSV-E7 was the most effective (80). Replication-defective alphavirus replicon particles have been used for HPV vaccination. A replicon particle vector of replication-defective Venezuelan equine encephalitis virus (VEE) containing HPV-16 E7 RNA was used for vaccination of mice and it enhanced CD8^+^ T-cell immune responses against HPV-16 E7 and eliminated established tumors (81). Replication-defective sindbis virus replicon particles encoding HPV-16 E7 fused to herpes simplex virus type 1 (HSV-1) VP22 induced improved CD8^+^ T-cell immune responses specific to HPV-16 E7 in vaccinated mice (82). Non-replicative pseudovirions are other tools of vaccination. Gene transfer with HPV pseudovirions showed more efficacy than DNA alone or liposome. In addition, HPV pseudovirions can generate mucosal and systemic E7-specific CTL responses (83). An important disadvantage of viral vectors includes safety issues (6). The advantage of viral vectors for development of HPV vaccines is their endogenously synthesis of HPV proteins from viral DNA by host cells and presented on the cell surface in conjunction with MHC class I molecules. Therefore, viral vaccines have no restriction on patient's HLA genotypes and can be administered in all subjects with different HLA types (28). The major concern for administration of viral vector for immunization is the production of antibodies against the viral vectors by host which may inhibit repetition of vaccination; therefore, the optimum immune responses against the desired antigen is expected to be achieved after the first dose of administered vaccine. Furthermore, safety concerns and pre-existing viral immunity in the recipient against viral vectors are other drawbacks (6, 28). As discussed before, the exposure of HPV antigens to immune system is limited; so, HPV viral vectors are appropriate vectors for development of new vaccines. 

 Some attenuated bacteria such as *Listeria monocytogeneses, Salmonella, Mycobacterium bovis, Shigella, *and* Escherichia coli *are used as bacterial vectors to deliver plasmids encoding the desired gene. Bacterial vectors can also deliver desired proteins to antigen presenting cells (APCs). Previous studies showed administration of an oral or intraperitoneal recombinant vaccine of *L.* *monocytogeneses* secreting HPV-16 E7 protein resulting in regression of E7-expressing murine tumors (84, 85). Bacilli Calmette-Guerin (BCG) was also used as a bacterial vector encoding HPV-16 L1 and E7 genes generating both E7-specific antibodies and cytotoxic immune responses (86). Applying the bacterial vectors for vaccination shows the same limitation as the viral vectors in production of antibodies against the vectors, safety concerns and pre-existing immunity in the recipient against the vectors. Specific antibodies against the bacterial vector should be checked before and after the first dose of the vaccine for achieving the appropriate immune responses in vaccinated subjects.

## Peptide/protein/DNA vaccines

Peptide-based vaccines are usually HLA-specific restricted in their usage for therapeutic vaccine strategies (6, 69). An HLA-A2-specific peptide vaccine consisting of 9 amino acids peptide from residues 12-20 of HPV-16 E7 protein was administered for women with HPV-16-associated disease; however, clinical results were not desirable (87). CTL epitopes of HPV-16 restricted to H-2D of murine and HLA-A2 of human was used for development of peptide-based vaccines for cervical cancer. Peptide vaccines showed minimal adverse side effects but they exhibit MHC restriction (69). In a study, vaccination with lipidated epitope of HPV-16 E7 induced CTL responses in some patients with HPV-associated cancers (88). Clinical data from another study demonstrated measurable enhancement in cytokine release and CTL responses in patients with HPV-16 and HLA-A2-positive high-grade cervical or vulvar intraepithelial neoplasia vaccinated with an E7-specific peptide-based vaccine (87). The most important limitation of the peptide-based vaccines is their specificity to certain HLA types; therefore, the administration for large population is limited. Selecting the specific immuno-dominant epitopes for the different HLA types is the main concern in these vaccines.

 In spite of peptide vaccines, protein vaccines are less dependent on HLA type of the patients (69). TA-GW, a fusion protein vaccine consisting of HPV-6 L2 and E7 proteins, is applied for treatment of genital warts (9, 89). Another fusion protein vaccine, TA-CIN vaccine, containing HPV-16 L2, E6 and E7 proteins generated E7-specific CD8^+^ T-cell immune responses in mice (90). Fusion of HPV proteins to heat shock proteins are the other kinds of HPV protein-based vaccines. HPV-16 E7 protein fused to HSP65 was administered in mice and led to regression of HPV-16 E7 expressing tumors in vaccinated animals (91). PD-E7 has been developed a mutated HPV-16 E7 linked to the first 108 amino acids of protein D of *Haemophilus influenza*e formulated in AS02B adjuvant. A small pilot study of the PD-E7 protein vaccine led to development of immune responses in vaccinated women (92). Although protein vaccines are almost safe, their production and purification process are difficult. Cold chain is needed for their storage and transport. Several vaccine doses should be administered for immunization. The type of adjuvant and amount of the protein in each dose of the vaccine may affect the immune responses in vaccinated subjects. In addition to possessing the high cost of the protein vaccines, these problems lead to examine other strategies include DNA vaccines. 

 DNA vaccines represent another approach to control of infectious agents (93). Compared to live viral or bacterial vectors, DNA vaccines have the advantages of easy production, storage and transport (15, 94). DNA vaccines generate continuously expression of desired antigen on MHC-peptide complexes in comparison with peptide or protein vaccines. In addition, the MHC restriction of peptide-based vaccines may be ignored (69). Furthermore, the naked DNA is relatively safe and can be repeatedly administered, since DNA vaccines do not induce antibody production against DNA in the vaccinated subjects (1, 95). Different methods can be used for administration of DNA vaccines such as intramuscular injection, intradermal injection (by needle or gene gun), intravenous injection and intranasal inoculation (69). DNA vaccines, however, are weakly immunogenic. Several strategies such as targeting DNA or encoded antigen to professional APCs may further the potency of DNA vaccines (1). Intracellular and intercellular targeting strategies are able to improve the MHC class I and/or II presentation of antigen (69). Several studies showed that linkage of HPV E7 to *M. tuberculosis* HSP70, calreticulin, *Pseudomonas aeruginosa* exotoxin A (domain II of its translocation domain), γ-tubulin, HSV-1 VP22 enhanced the immune responses against desired gene in administered-DNA vaccine (96-100). Our previous study proved that administration of a DNA vaccine containing immunogenic region of human papillomavirus type 16 E7 gene linked to human HSP70 gene was able to induce the CMI responses in mice (94). 

 Another strategy for enhancing the HPV DNA vaccine potency is co-administration of the E7 DNA vaccine with a DNA containing anti-apoptotic proteins such as Bcl-xL, Bcl-2, X-linked inhibitor of apoptosis protein, dominant negative caspase-9 and/or dominant negative caspase-8. The anti-apoptotic proteins are able to improve E7-specific immune responses and resulted in regression of tumor and DC survival (101). Co-administration of E7 DNA vaccine with cytokines or co-stimulatory molecules and codon optimization of E7 for increasing the antigen presentation are other strategies used for improving the immune responses against E7 DNA vaccine in vaccinated subjects (102-105). A DNA vaccine containing HLA-A2-restricted epitopes of HPV-16 E7, ZYC101, was administered in patients with high-grade anal intraepithelial lesions. Their primary results showed that the vaccine was well tolerated in all vaccinated subjects and the immune responses were increased against the epitopes encoded by the vaccine (106). In preclinical studies, another DNA vaccine of similar chain PADRE (invariant Pan HLA-DR reactive epitope) was administered and showed both preventive and therapeutic effects (107). A novel DNA vaccine encoding HPV-16 E7 protein was designed. The strategy was expression of the HPV E7 protein in the endosomal compartment. The vaccine generated CD4^+^ T-cell dependent protection in mice (108). One of the DNA vaccine disadvantages is the possibility of integration of DNA vaccine into the host genome that could lead to activation of proto-oncogenes or inactivation of tumor suppressor genes. Another concern of DNA vaccines is production of antibodies against DNA that lead to autoimmune disease (109-111).

## Other strategies

DC-based vaccines are another strategy for developing the therapeutic vaccines against HPV-associated tumors. In this strategy, DCs were pulsed with E6 and/or E7 peptides/proteins. In DC-based vaccines, the route of administration of the vaccine affects the efficacy of the vaccine (69).

 Chimeric HPV VLP vaccines are another method for HPV vaccine development. HPV VLPs of L1 or L1/L2 can be fused to a foreign epitope or polypeptides. Several studies showed chimeric VLPs encoding E2 and/or E7 antigen induces both neutralizing antibodies and T-cell mediated immune responses (48, 112). Such vaccines act as both preventive and therapeutic vaccines to protect individuals from HPV infections and to treat the established HPV infections, HPV associated diseases and cancers. A chimeric vaccine of VLP and E2 was administered in mice and strong immunogenic responses to both L1 and E2 were generated in vaccinated animals (113). A chimeric VLP vaccine of HPV L1-E7 was administered to target HPV-16-associated high-grade squamous intraepithelial lesions. Unfortunately, the results showed no statistical effect on regression (6). Another study showed that administration of a chimeric VLP vaccine encoding HPV-16 E7 was able to generate T-cell responses to E7 (112). Such strategies may be useful in development of HPV vaccines with both therapeutic and preventive effects. In another clinical trial, a chimeric fusion protein containing L2, E6 and E7 of HPV-16 which does not form VLPs, was used for vaccination. Their results showed no clinical effects on CIN regression (90).

 Prime-boost regimen is another strategy in HPV vaccination. In a study, various combinations of viral vectors and nucleic acids were tested in a prime-boost regimen and suggested that priming with a DNA vaccine followed by a recombinant vaccinia viral vector booster might provide the most anti-tumor effects (114). In another study, priming with L1 expressing DNA and boosting with adenoviral vector encoding HPV-16 L1 induces antibodies especially IgG2a and IgG2b isotypes in sera of vaccinated mice (115).

## Conclusion

VLP-based preventive vaccines opened a prospective path for prevention of benign and malignant anogenital diseases caused by more prevalent genital HPV types. Two prophylactic vaccines against HPVs have been developed. Gardasil® is a quadrivalent vaccine consisting L1 proteins of HPV types 6, 11, 16 and 18. Another vaccine is Cervarix™ that is a bivalent vaccine containing L1 proteins of HPV types 16 and 18. Both preventive vaccines, Gardasil® and Cervarix™, are in the last stages of their clinical trials and their previous results demonstrated that they are safe, well tolerated and are able to generate high titers of neutralizing antibodies against vaccines' HPV types; however, they do not cover all high-risk papillomaviruses that cause cervical cancer. They can protect the body against only 70% of the HPV associated cervical cancers. Designing the other HPV VLP-based vaccines will be required for protection against a broad range of HPV high-risk types that cause cervical cancer. Therefore, current preventive vaccines will be able to reduce but not eliminate the risk of cervical cancers and other premalignant diseases related to HPVs. Although the prophylactic vaccine protection persists for at least 5 years, there is also a need to define duration of the vaccine protection. Furthermore, their safety in pregnant women is an issue for future studies. Another limitation for HPV preventive vaccines is their efficacy in different countries. In some countries with high rate of HPV associated diseases, the predominant high-risk HPV types may be different. Furthermore, it is possible that the elimination of common HPV types by prophylactic vaccination leads to an increase in the risk of infection with other high-risk types of HPV that are not included in the vaccines, resulting the increase in HPV related diseases and cancers in long time. 

 In addition to prophylactic vaccines, developments of therapeutic HPV vaccines are required for treatment of women who are currently infected with HPVs and require the treatment. Several strategies including vector-base vaccines, protein-based vaccines, peptide-based vaccines, nucleic acid-based vaccines, cell-based vaccines and chimeric vaccines enhance the virus-specific cell-mediated immunity activation and anti-tumor effects in mice tumor systems. Most of the HPV therapeutic vaccines are developed for targeting HPV E6 and/or E7; however, they have not shown the same success as the prophylactic vaccines in their clinical trials. The comparison of these methods will single out the most potent therapeutic vaccines with the minimum level of negative side effects against HPV established infections and cervical cancer.
